# A mutated rabbit defensin NP-1 produced by *Chlorella ellipsoidea* can improve the growth performance of broiler chickens

**DOI:** 10.1038/s41598-019-49252-4

**Published:** 2019-09-04

**Authors:** Chengming Fan, Jihua Wu, Ling Xu, Lili Bai, Heming Yang, Congjuan Yan, Qing Wu, Yuhong Chen, Zanmin Hu

**Affiliations:** 1 State Key Laboratory of Plant Cell and Chromosome Engineering, The Innovative Academy of Seed Design, Institute of Genetics and Developmental Biology, Chinese Academy of Sciences, Beijing, 100101 China; 2 The 306th Hospital of PLA, Beijing, 100101 China; 3 Polo Biology Science Park Co., Ltd., Beijing, 101113 China; 4 School of Food and Biological Engineering, Jiangsu University, Zhenjiang, 212013 China; 5 College of Life Sciences, University of Chinese Academy of Sciences, Beijing, 100049 China

**Keywords:** Molecular engineering in plants, Animal physiology

## Abstract

The demand for alternatives to antibiotics to improve the growth performance of food animals is increasing. Defensins constitute the first line of defence against pathogens in the innate immune system of animals and humans. A transgenic *Chlorella ellipsoidea* strain producing mNP-1 (a mutated rabbit defensin NP-1) was previously obtained in our laboratory. In this study, a process for producing the transgenic strain on a large scale was developed, and the *C*. *ellipsoidea* strain producing mNP-1 was used as a feed additive to improve the health and growth performance of chickens. The volume of *C*. *ellipsoidea* producing mNP-1 can be scaled up to 10,000 L with approximately 100 g/L dry biomass, and the mNP-1 content of transgenic microalgal powder (TMP) was 90–105 mg/L. A TMP-to-regular feed ratio of 1‰, as the optimal effective dose, can promote the growth of broiler chickens by increasing weight by 9.27–12.95%. mNP-1 can improve duodenum morphology by promoting long and thin villi and affect the microbial community of the duodenum by increasing the diversity and abundance of beneficial microbes. These results suggested that transgenic Chlorella producing mNP-1 can be industrially produced and used as an effective feed additive and an alternative to antibiotics for improving the health and growth performance of broiler chickens or other types of food animals/poultry.

## Introduction

Antibiotics have traditionally been used as growth promoters at sub-therapeutic levels to enhance food animal growth performance and stabilize their health status^1,2^; however, antibiotic-resistant microorganisms occur in food-producing animals fed with growth-promoting levels of antibiotics and can be transmitted from animals to the human microbiota^3^. The overuse of antibiotics has increasingly been highlighted as a result of increasing consumer awareness, and there is increasing demand for livestock products from antibiotic-free production systems^2^. To avoid the transfer of resistance genes from animals to human pathogens, which represents a potential threat to human health, the European Commission first decided to ban the use of common feed antibiotics as growth promoters in feed beginning in 2006, and feed antibiotics are now being phased out in many states^4^. Since 2006, the use of antimicrobial peptides (AMPs), probiotics, prebiotics, synbiotics, (in)organic acids, enzymes, phytogenics, hyperimmune egg antibodies, bacteriophages, clay and metals as alternatives to the use of antibiotics as growth promoters has attracted more attention in food animal production^2^. AMPs target a broad spectrum of organisms with antibiotic resistance, such as bacteria, yeasts, fungi, viruses or parasites^2^, and are also referred to as ‘host defence peptides’, emphasizing their additional immunomodulatory activities in higher eukaryotic organisms^5,6^. The activities of AMPs are diverse, are specific to the type of AMP, and include a variety of cytokine- and growth factor-like effects that are relevant to normal immune homeostasis^5^.

Defensins, one group of AMPs, are small, multifunctional cationic peptides and typically contain six conserved cysteines that form three intramolecular disulphide bonds stabilizing a primarily β-sheet structure that is composed of α-, β- and θ-defensin subfamily proteins in vertebrate animals^7^. However, some cysteine-rich AMPs in plants, fungi, myxobacteria and invertebrates are also called defensins. Defensins are involved in the first line of defence in the innate immune response against pathogens such as bacteria, fungi and viruses^7–9^. Defensins may localize on bacterial membranes through electrostatic steering and then disrupt membranes through several mechanisms, including the formation of carpets, pores and general membrane instability^9^, which may help to reduce the emergence of resistant strains. Defensins are therefore a potential source of a new class of drugs^8,10^. Rabbit neutrophil peptide-1 (NP-1), a member of the α-defensin subfamily, shows good biological activity against pathogens^11–14^ and in sciatic nerve regeneration^15^.

Microalgae are unicellular eukaryotic organisms with a high growth rate, low growth costs, a short culture period and metabolic pathways similar to those of higher plant cells. They are regarded as a new bioreactor that is becoming more attractive in the production of biologically active substances, such as vaccines, antibodies, enzymes, blood-clotting factors, immune regulators, growth factors and hormones^16^.

Our previous study showed that mNP-1 (a mature NP-1 with an additional methionine at the N-terminus) can be produced in *Chlorella ellipsoidea*^17^ at a concentration of 11.42 mg/L in flasks. In this study, industrial preparation of mNP-1 was carried out by culturing the mNP-1 transgenic *C*. *ellipsoidea* in a 10-m^3^ fermenter. Then, transgenic microalgal powder (TMP) was used as a feed additive to promote the health of animals with the goal of replacing or reducing antibiotic use in the process of raising broiler chickens. In addition, the mechanism by which mNP-1 improved chicken health was further investigated by histological dissection and 16S rRNA-based analysis of the gut microflora.

## Results

### The technology for industrial mNP-1 preparation was developed by large-scale culturing of transgenic *C*. *ellipsoidea* expressing mNP-1

**Figure 1:**
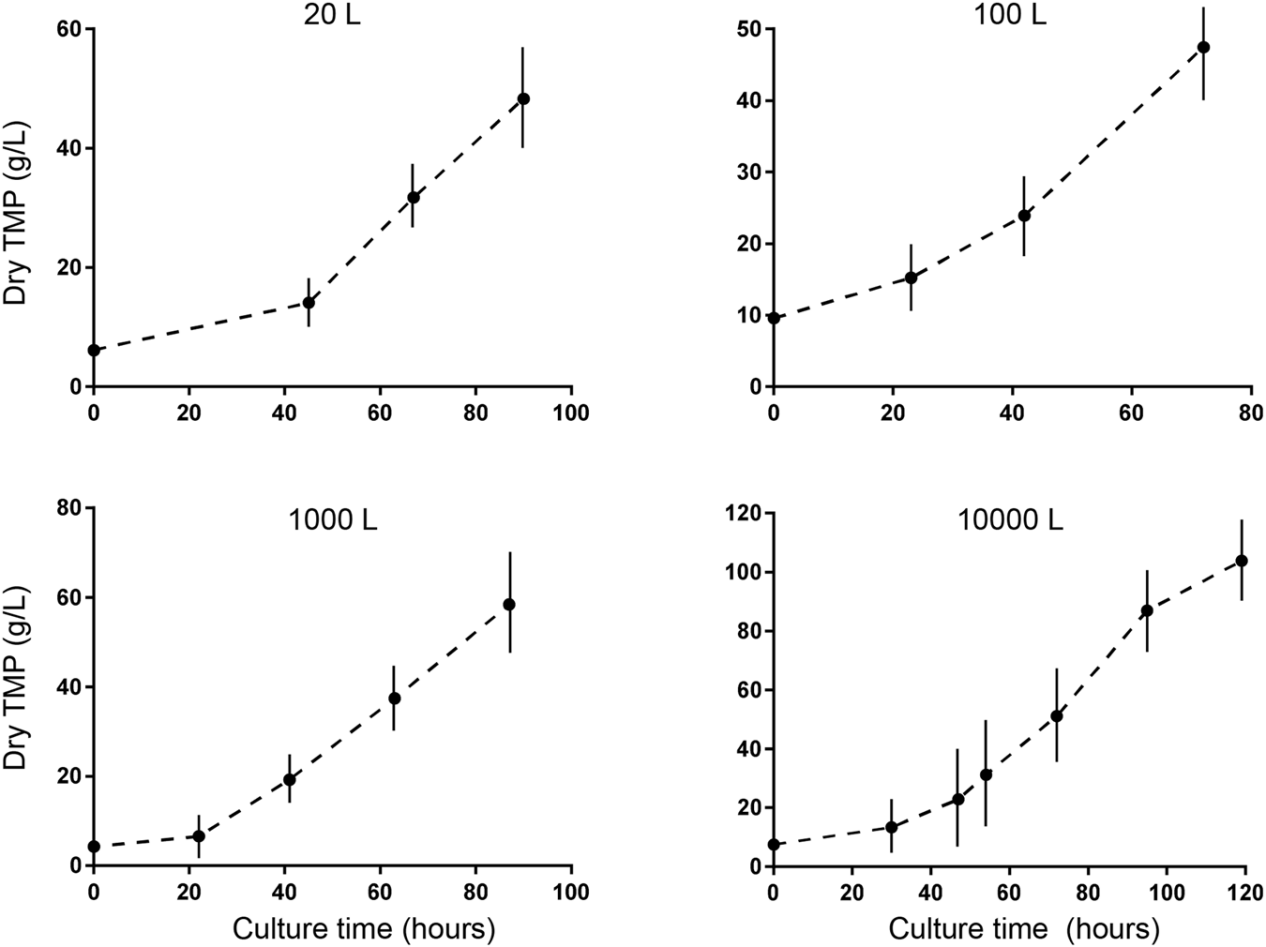
Industrial preparation of TMP in a 10,000 L fermenter. The growth of the mNP-1 transgenic *C*. *ellipsoidea* cultures in 20 L, 100 L, 1000 L and 10000 L fermenters was quantified at different culture times; data are shown as the mean +/− SD, n = 3.

The industrial preparation of mNP-1 was completed through batch fermentations. At the optimal ratio of glucose to urea (20:1), the pH of the culture broth was maintained in the range of 6.8 to 7.5 by the fed-batch culture method without the addition of acid or base. The transgenic *C*. *ellipsoidea* seeds were successively cultured in 500 mL and 3 L-flask cultures (OD, approximately 2.0) and then scaled up from 20 L to 100 and 1000 L fermenters (Fig. 1). After that, the transgenic *C*. *ellipsoidea* strain was cultured in a 10-m^3^ fermenter, and a dry biomass of 90–105 g/L (dTMP) was achieved (Fig. 1).

### mNP-1 can enhance the growth performance of broiler chickens

**Figure 2:**
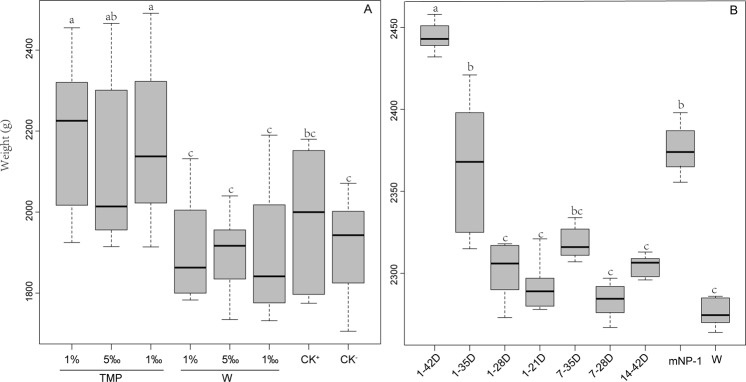
TMP improved the growth performance of broiler chickens. (**A**) Identification of the optimal effective dose of TMP. The concentrations of 1‰, 5‰ and 1% TMP or W in the feed were tested. CK^−^, base diet; CK^+^, base diet with 15 mg/kg virginiamycin. (**B**) Screening the suitable feeding model. TMP (1‰) was added to the feed at different time points in the experiment: 1–42D, 1–35D, 1–28D, 1–21D, 7–35D, 7–28D, 14–42D, in which the first number is the starting day and the second number is the ending day. W, wild-type microalgal powder. mNP-1, the treatment with crude mNP-1 peptides added to the water at a concentration of 0.5 mg/L. Data are shown as the mean +/− SD, n = 5. The letters on the bar indicate the significance of the difference at p < 0.05.

Compared with the negative treatment (CK^−^), the weights of the broiler chickens supplemented with 1‰, 5‰ and 1% TMP increased by 12.95 ± 1.33%, 9.93 ± 1.07% and 14.22 ± 1.40% (p < 0.05), respectively, and the chickens showed no other significant differences at the p < 0.05 level at 42 days. However, the three TMP-treated groups gained significantly more weight than the birds fed the same concentration of wild-type microalga powder or virginiamycin (Fig. 2A). The weight of the birds increased by 3.89 ± 0.39% in the positive control, but there were no differences compared with the negative control. Based on the above results, 1‰ TMP can be selected as the optimal effective dose to promote the growth of broiler chickens.

mNP-1 crudely extracted from the transgenic *C*. *ellipsoidea* strain can also significantly improve the growth of broiler chickens. Crude mNP-1 peptides (more than 95% purity) were extracted from TMP and added to the water (0.5 mg/L, equivalent to 1‰ TMP in feed, because water intake is approximately 2 times that of feed intake), and the birds drank freely for 42 days. The average weight of the birds treated with mNP-1 was 2368.00 ± 30.84 g and increased by 4.06 ± 0.04% compared with wild-type microalgal powder (Fig. 2B).

After identifying the optimal effective dose of TMP, the effects of seven TMP groups were determined to identify the suitable feeding model. The results (Fig. 2B) revealed that the base diet with 1‰ TMP fed for different periods can increase the weight of the birds by 5.37 ± 0.12% to 12.79 ± 0.28%. The group fed TMP for 42 consecutive days exhibited the most significant weight gain compared to the other groups at the p < 0.01 level. However, the weights were not significantly different among the 1–28D, 1–21D, 7–35D, 7–28D, 14–42D and W groups. Our results revealed that feeding the base diet supplemented with 1‰ TMP could increase the weight of broiler chickens and that feeding TMP for 42 consecutive days could result in the largest weight gain.

### mNP-1 can improve the villus morphology of the duodenum

**Figure 3:**
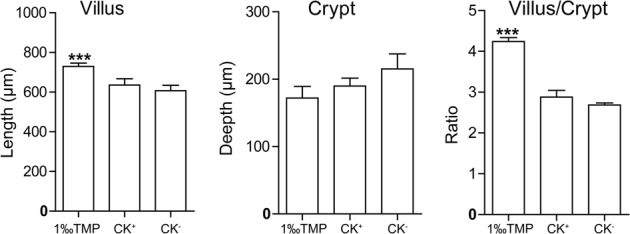
Changes in duodenum villi and crypts. 1‰ TMP, base diet with 1‰ TMP. CK^−^, base diet. CK^+^, base diet with 15 mg/kg virginiamycin. Ratio, the average ratio of the average length of the villus to the average depth of the crypt in one treatment. Data are shown as the mean +/− SD, n = 10. ***Significantly different compared with the CK^−^ at p < 0.01.

To identify the possible reasons for the weight gain of broiler chickens induced by feeding mNP-1, the routine blood and biochemical indexes of the treated chickens on day 42 of the experiment were first examined; however, no differences were found among the different treatments (data not shown). Sections of tissues such as the heart, liver, lungs, kidneys, thymus, pancreas, jejunum and ileum were then histologically tested, and they showed no significant differences among the treatment groups (data not shown). However, in the duodenum, the length of the villi was significantly increased (p < 0.01) (Fig. 3). Intestinal villi are the main organs involved in nutrient absorption, and the increase in villi length suggested that the efficiency of feed absorption was improved.

### mNP-1 affected the microbial community of the duodenum

**Figure 4:**
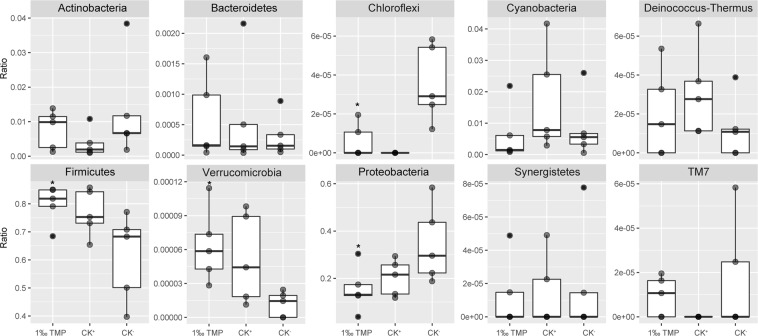
Changes in the microbial community of the duodenum at the phylum level. Changes in the microbial community of the duodenum of the tested chickens at the phylum level were calculated. 1‰ TMP, base diet with 1‰ TMP. CK^−^, base diet. CK^+^, base diet with 15 mg/kg virginiamycin. One grep dot shows the average proportion of one phylum compared to all phyla in one group. Each treatment had five replicate groups, and every group had 2 samples. Data are shown as the mean +/− SD, n = 5. *There was a significant difference between 1‰ TMP and CK^−^ at p < 0.05.

Because the villi height changed in the duodenum, the duodenum microbial community was investigated using 16 S rDNA sequences in the 1‰ TMP, CK^+^ and CK^−^ groups. According to the results, ten known bacterial phyla (Fig. 4) (Firmicutes, Proteobacteria, Actinobacteria, Cyanobacteria, Verrucomicrobia, Bacteroidetes, Deinococcus-Thermus, Chloroflexi, Synergistetes, and TM7) and one unknown phylum were detected (Supplementary Table S1). All ten known bacterial phyla were detected in the 1‰ TMP and CK^−^ groups, while Chloroflexi and TM7 were not detectable in the CK^+^ group (Fig. 4). Compared with those in the CK^−^ group, the levels of Firmicutes and Verrucomicrobia significantly increased, while the levels of Proteobacteria and Chloroflexi significantly decreased in the 1‰ TMP group at p < 0.05 (Fig. 4).

Firmicutes and Proteobacteria were the dominant bacteria in the chicken duodenum and were significantly different (p < 0.05) among the three treatments. The level of Firmicutes was 0.6119 ± 0.1565, 0.6929 ± 0.1447 and 0.7988 ± 0.0686 in the CK^−^, CK^+^ and 1‰ TMP groups, respectively, and the level of Proteobacteria was 0.3456 ± 0.1639, 0.2038 ± 0.0764 and 0.1547 ± 0.0970 in the CK^−^, CK^+^ and 1‰TMP groups, respectively.

**Figure 5:**
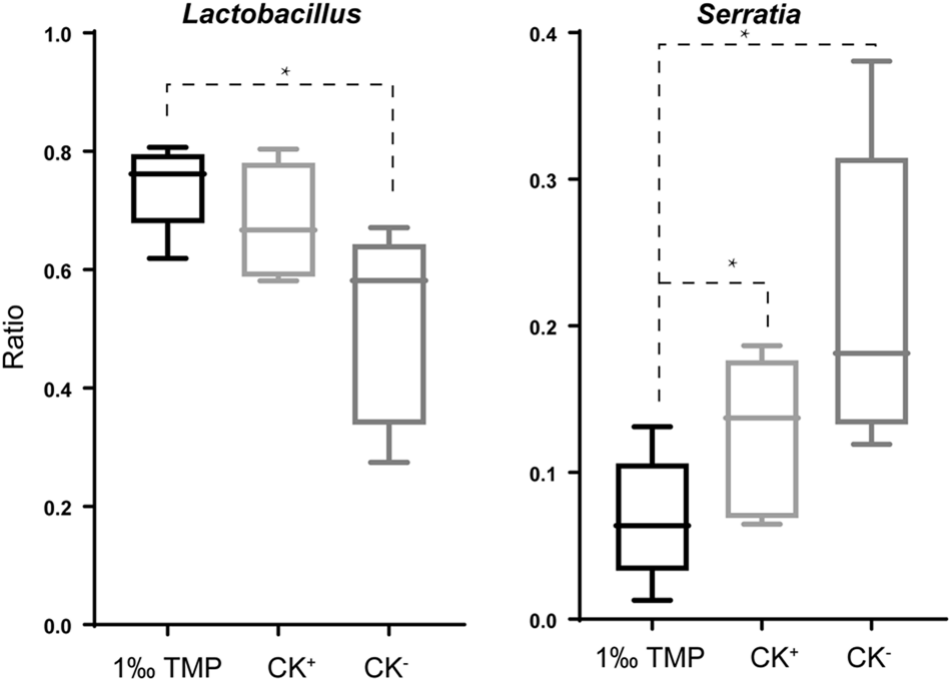
Changes in *Lactobacillus* and *Serratia* in the duodenum. 1‰ TMP, base diet with 1‰ TMP. CK^−^, base diet. CK^+^, base diet with 15 mg/kg virginiamycin. Ratio, the average proportion of one genus among all genera in one treatment. Each treatment had five replicate groups, and every group had 2 samples. Data are shown as the mean +/− SD, n = 5. *Significant difference at p < 0.05.

In all the samples, approximately 112 genera were identified, and 92, 87 and 102 genera occurred in the CK^−^, CK^+^ and 1‰ TMP groups, respectively (Supplementary Table S2). Only ten genera were detected in the 1‰ TMP group, and only 12 genera were detected in the CK^−^ and 1‰ TMP groups (Supplementary Table S2). The results showed that the microbial community in the duodenum has more biodiversity after the 1‰ TMP treatment than after the other two treatments. In all the genera, *Lactobacillus*, a class of probiotics, was the dominant bacterium, and the ratios were 0.5087 ± 0.1653, 0.5781 ± 0.1758 and 0.7418 ± 0.0732 in the CK^−^, CK^+^ and 1‰ TMP groups, respectively (Fig. 5 and Supplementary Table S2). *Serratia*, a pathogen, was the second dominant genus, with levels of 0.2153 ± 0.1044, 0.1622 ± 0.0699 and 0.0685 ± 0.0432 in the CK^−^, CK^+^ and 1‰ TMP groups, respectively, and there were significant differences (p < 0.05) among them (Fig. 4D).

**Figure 6:**
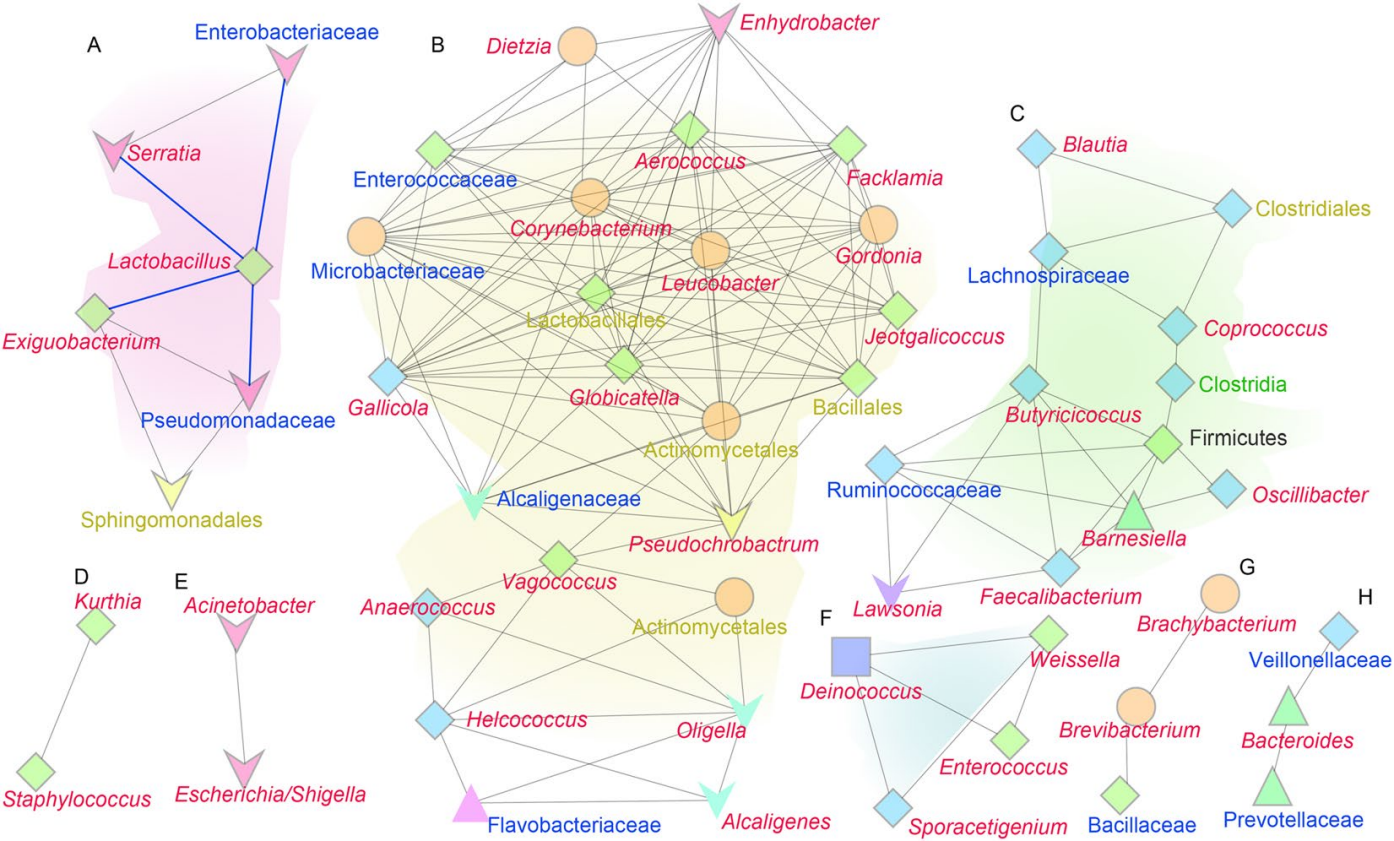
Co-occurrence relationships of different genera in the duodenum. The co-occurrence relationship of bacterial genera in the duodenum samples was constructed using Cytoscape v3.5.1^40^. (**A**–**H**) The groups of bacteria with absolute values of correlation coefficients greater than 0.85 and p-values less than 0.05. Different shapes indicated different bacterial phyla, different fill colours of the shapes indicate different bacterial classes, blue lines indicate correlation coefficient values less than −0.85, and other lines indicate correlation coefficient values greater than 0.85. The words in red, blue, yellow, green and black indicate the classifications that could be identified in this study at the genus, family, order, class and phylum levels, respectively.

To identify the co-occurrence of bacteria in the duodenum, the correlation coefficients of 74 common bacteria were calculated at the genus level in all duodenum samples. According to the results, the possible co-occurrence relationships of 56 genera, in which the absolute values of the correlation coefficients (Supplementary Table S3) were more than 0.85 and the p-values were less than 0.05, were constructed and classified into 8 groups: A to H (Fig. 6). Except for group A relative to *Lactobacillus*, the ratios of the members of other groups were less than 0.1% in the 3 treatments (Supplementary Table S2), so they may not play important roles in improving the health and growth performance of the broiler chickens. Group A, including Firmicutes (*Lactobacillus* and *Exiguobacterium*) and Proteobacteria (*Serratia* and other 3 unknown genera), was the dominant microbe group according to the above results (Fig. 5, Supplementary Table S2). *Lactobacillus* and *Serratia* were the dominant microbes in the three treatments, and there was a significant negative correlation between them (correlation coefficient = −0.95, p-value = 6.69E-8).

## Discussion

Defensins are an important family of AMP peptides that could be used in many fields, such as pharmaceutics and agriculture. However, the production of active defensins has been hampered due to the lack of appropriate expression systems^18^. The main hosts used for the heterologous expression and production of defensin are bacteria, yeasts and plant^18^. However, the final yield or bioactivity of defensins is low because of the toxicity of AMPs towards host cells, including peptide degradation and protein misfolding or modification in bacteria and yeasts^16,18,19^. Although some reports have claimed to produce large amounts of β-defensins in bacteria, they have failed to prove the correct folding and activity of such antimicrobial peptides^18,19^. In *Pichia pastoris*, the yields of recombinant defensins are 7–165 mg/L^20^. Most of the studies on defensin production are still performed in shake flasks^19,21^. However, a study showed that approximately 165.0 mg/L recombinant HD5 mature peptide cultured under optimal conditions in a 15-L bioreactor can be obtained after a 48 h incubation, and approximately 50% of the initial recombinant HD5 mature peptide was recovered after purification without folding or modification, with prominent antibacterial activity and the ability to block human papillomavirus infection^19,22^. In *C*. *ellipsoidea*, the production of mNP-1 is approximately 11.42 mg/L under shake flask culture conditions^23^. In this study, under heterotrophic culture conditions, *C*. *ellipsoidea* was used as a bioreactor for the industrial preparation of mNP-1. According to our strategies, the fermentation process was scaled up, and a dry biomass of 103 g/L was achieved for the culture of transgenic *C*. *ellipsoidea* in a 10-m^3^ fermenter (Fig. 1). The content of mNP-1 in the dry TMP was approximately 100 mg/g. The achievement of mNP-1 industrial preparation will enhance the application value of mNP-1.

The main benefits of antibiotic feed additives are increasing productivity and improving animal health^1,24^. Because of the potential adverse effects of sub-therapeutic doses of antibiotic additives in animal feed on human health, food safety and the environment, complete bans or restricted use regulations have been implemented in some countries, and the quest for alternative products or approaches has intensified in recent years^1,2^. Much research has been carried out to discover alternatives to antibiotics that have beneficial effects similar to growth promoters^25^. For example, one important and potential alternative product is AMP, which is a diverse class of naturally occurring molecules that are produced as a first line of defence by all multicellular organisms^5^. Many studies have been conducted on AMPs, and their applications in poultry have mostly focused on their protective potential against diverse pathogens causing infectious diseases rather than their growth-promoting activities^2^. However, the effects of a few AMPs on poultry growth performance have been investigated^26–28^. For example, cecropin A(1–11)-D(12-37)-Asn (CADN, a chimeric peptide derived from insects) supplementation at the optimum dose can increase broiler weight gain, feed intake, the feed/gain ratio, and intestinal villus height while decreasing aerobic bacterial counts in both jejunal and caecal digested matter^26^. The AMPs extracted from the swine gut^27^ and the rabbit sacculus rotundus^28^ can also improve the growth performance, intestinal morphology and health of broilers. Defensins are a group of AMPs with six conserved cysteine residues that form intramolecular disulphide bonds and play important roles in the innate immune response against infectious pathogens. However, until now, there has been no report that defensins could be used as feed additive. In this study, we first revealed that the use of mNP-1 as a feed additive can increase the production performance and improve the health of broiler chickens. Supplementation of the base diet of broiler chickens with TMP including mNP-1 significantly increased weight gain (Fig. 2A) compared with the control. In addition, mNP-1 can increase villus height, similar to cecropin^26^. Thus, mNP-1 has growth-promoting activity and represents a new alternative to antibiotics in feed additives.

Several ideas have been proposed to elucidate the mechanism behind antibiotic-mediated growth enhancement, but to date, there is no clear explanation^2,25^. However, antibiotics are natural metabolites of fungi that inhibit the growth of bacteria. Thus, antibiotics used as feed additives at sub-therapeutic doses can remodel microbial diversity and relative abundance in the intestine to provide an optimal microbiota for growth or health^3,25^. Virginiamycin can significantly improve the growth performance of broilers^29^, and the use of virginiamycin (100 ppm) as a growth promoter has been associated with an increased abundance of *Lactobacillus* species in the broiler duodenal loop in the proximal ileum^30^. NP-1 is a multifunctional cationic peptide with broad-spectrum antimicrobial activities^11–13^. For example, the purified recombinant rabbit NP-1 from *Escherichia coli* showed significant antimicrobial activity against clinically relevant bacteria, including *E*. *coli* and *Pseudomonas aeruginosa* (gram negative) and *Staphylococcus aureus* and *Bacillus subtilis* (gram positive)^11^. The isolated rabbit peptide NP-1 from rabbit neutrophils can kill oral, gram-negative, facultative bacteria, including *Actinobacillus actinomycetemcomitans*, *Eikenella corrodens*, and *Capnocytophaga* spp^13^. In addition, NP-1 can directly inactivate some viruses^14^. The use of TMP with mNP-1 as a feed additive can increase the abundance of *Bifidobacterium* and *Lactobacillus* in the rat gut microbiota^31^. Our results revealed that the bacterial diversity at the genus level of chicken duodenums in the group treated with 1‰ TMP was greater than that in the other groups (Fig. 4 and Supplementary Table S2). In the 1‰ TMP group, 102 genera were detected in the chicken duodenum, ten of which were unique. Additionally, TMP can significantly increase the level of the probiotic *Lactobacillus*, and virginiamycin (CK^+^) can also increase the level of the probiotic *Lactobacillus* (p-value = 0.54) (Fig. 5). TMP and virginiamycin (CK^+^) significantly reduced the pathogenic bacterium *Serratia* (Fig. 5). Additionally, according to the co-occurrence relationship analysis, a significant negative correlation occurred between *Lactobacillus* and *Serratia*. Therefore, the dynamic balance between *Lactobacillus* and *Serratia* may have important roles in maintaining the health of boiler chickens. Our results are consistent with a previous report showing that the direct feeding of *Lactobacillus* sp. or their culture could improve broiler productive performance and metabolic functions, promoting animal health^32–34^. Therefore, one reason to use m-NP-1 as a feed additive to improve chicken health could be that m-NP-1 can increase duodenal *Lactobacillus* and adjust the balance and quality of the intestinal microflora. However, the mechanism by which mNP-1 affects intestinal microbial diversity is not clear. We hypothesized that mNP-1 may affect the intestinal microbial diversity of chickens through the innate immune response of chickens because it is a component of the innate immune system, defending against pathogens in different living organisms^6–8^.

Cost is one of the major bottlenecks in the application of microalgae and defensin technology. In this study, the optimal ratio of glucose to urea was developed, and the pH value of the culture broth was stably controlled in the range of 6.8 to 7.5 by the fed-batch culture method. This strategy provided good control of the pH without addition of acid or base. It simplified the fermentation process and reduced production costs. To date, there have been no reports that defensin can be industrially produced. In this study, mNP-1 can be produced in a 10,000 L fermenter with approximately 0.1 g/L, and TMP production was up to 100 g/L. According to the optimal dosage used in this study, the TMP supplemental diet is cost affordable. It would be more economical if TMP were used for highly valuable animals, birds and aquatic animals, which needs to be further investigated.

Microalgae can provide many important bioactive molecules, such as vitamins, essential amino acids, polyunsaturated fatty acids, minerals, carotenoids, enzymes and fibre, for food and feed^35^ and have many advantages for the production of specific molecules^35,36^. With significant advances in microalgae biotechnology over the last decade, valuable products, such as vaccines, antibodies, enzymes, blood-clotting factors, immune regulators, growth factors, and hormones, have been developed via the use of microalgae as bioreactors^16^. In this study, our results suggested that *C*. *ellipsoidea*, a valuable unicellular algae, can be used as an effective bioreactor to produce the defensin mNP-1 on a large scale and may be used to produce other bioactive materials for direct application (without purification) in food animal production and human health.

## Methods

### Industrial production of transgenic microalgae

*mNP-1* encoding a mature α-defensin with 34 aa (M added at the N-terminal peptide of the mature NP-1 from rabbit neutrophilic granulocytes) was expressed in the microalgal bioreactor *C*. *ellipsoidea*^23^. For industrial preparation, the following medium components were used to culture the transgenic *C*. *ellipsoidea*: 30 g/L glucose, 1.5 g/L urea, 1.2 g/L KH_2_PO_4_, 1.2 g/L MgSO_4_·7H_2_O, 0.2 g/L sodium citrate, 0.105 g/L CaCl_2_·2H_2_O, 0.016 g/L FeSO_4_·7H_2_O, 0.002 g/L EDTA, and a mixture of microelements (2.86 mg/L H_3_BO_3_, 1.81 mg/L MnCl_2_·4H_2_O, 0.222 mg/L ZnSO_4_·7H_2_O, 0.021 mg/L NaMoO_4_ and 0.07 mg/L CuSO_4_·2H_2_O). The other major fermentation parameters included maintaining a temperature of 25 ± 0.1 °C, pH of 6.8–7.2, dissolved oxygen of ≥50%, and pressure of 0.03–0.05 MP. When the glucose content dropped below 5 g/L, more was added, and the highest concentration of glucose was 30 g/L. The step-by-step increases in the volume of the fermenters were 20, 100, 1000 and 10000 L. The extraction and determination of crude mNP-1 peptides were performed according to Bai *et al*.’s methods^17^. TMP was obtained using the spray drying method according to the manual of the spray dryer LGZ300 (Wuxi Dongsheng Spray-Granulating and Drying Equipment Factory, Wuxi, China).

### Bird rearing

The experimental protocols applied in this study complied with the guidelines for animal welfare and were approved by the Animal Care and Use Committee of the Institute of Genetics and Developmental Biology, Chinese Academy of Sciences (AP2016056), and all efforts were made to minimize suffering.

The broiler chicken “Baiyu AA” was selected to evaluate the effectiveness of TMP as a feed additive. One-day-old birds were randomly grouped into different pens of identical size. The temperature, feed, immunization, and regular management followed references^37^. The feed was provided by Beijing Dafa Zhengda Co. Ltd. and was specific for “Baiyu AA”. Every pen had the same number of male and female birds. Birds were weighed once a week, and the performance of 42-day-old birds was used to analyse the effect of TMP. In the following feeding experiments, every treatment had 5 replicates of 5 male and 5 female birds each. The group fed the base diet was used as the negative control, and the group treated with added antibiotic (virginiamycin, 15 mg/kg) was used as the positive control.

To identify the optimal effective dose of TMP, six treatments were administered as follows: the base diet containing 1‰, 5‰ or 1% TMP or base diets containing 1‰, 5‰ or 1% wild-type microalgal powder as the corresponding mock control over the 42-day feeding process.

After the optimal dose was added, the suitable feeding administration was investigated. TMP was added to the diet on the following schedules: from day 1 to day 21 28, 35, or 42; from day 7 to day 28 or day 35; and from day 14 to 42. The negative control birds were fed the base diet. In addition, the dose of wild-type microalgal powder used as the mock control was added for 42 days and was equivalent to the optimal dose of TMP added.

### Sample collection

To investigate the possible mechanism underlying the mNP-1 peptide-mediated enhancement of the growth of broiler chickens, organ coefficients, tissue sections and routine blood and biochemical indexes were examined. One female and one male bird in each replicate with live weights close to the mean were immediately killed by cervical dislocation at the end of the treatment period. The internal organs, such as the heart, liver, lungs, kidneys, thymus and pancreas, were quickly weighed to calculate the percentages of live weight (organ coefficient) and then fixed in a 4% formaldehyde solution for pathological and histomorphological analysis. Afterwards, the same size (approximately 2.0 cm) and position of the duodenum, jejunum and ileum of each bird were washed with 0.75% NaCl solution and fixed with a 4% formaldehyde solution for the analysis of variable intestinal villus morphology^38^. The duodenum digesta of two birds were collected into 10 mL sterile tubes, which were stored first in liquid nitrogen and then at −80 °C for the 16S rRNA-based analysis of the gut microflora.

Blood samples were collected (3–5 mL) from the wing veins of the selected birds into a 10-mL anticoagulant-free vacutainer tube. Then, the tubes were incubated at approximately 30 °C for approximately 30–60 min, and the serum was collected after centrifugation for 10 min (at 1,500 × g at room temperature) for blood biochemical analysis. For example, the concentrations of serum IgG, IgA, and IgM were assayed using appropriately diluted samples by a sandwich ELISA with chicken-specific IgG (Cat. No. E30–104), IgA (Cat. No. E30–103), and IgM (Cat. No. E10–101) ELISA quantitation kits (Bethyl Laboratories Inc., Montgomery, TX) according to the manufacturer’s instructions. The routine blood and biochemical indexes were determined by the Beijing Sino-UK Institute of Biological Technology. Histological sectioning and haematoxylin-eosin staining were carried out at the 306th Hospital of PLA^39^.

### Total DNA extraction from intestinal microorganisms and 16S rDNA analysis

Microbial genomic DNA was extracted from faecal samples using a QIAamp DNA stool mini kit (QIAGEN, cat#51504) following the manufacturer’s recommendations and was used to evaluate the effect of TMP on intestinal microorganisms. The 16S rRNA-based analysis of the gut microflora was conducted by Guangzhou RuiBo Biotechnology Co., Ltd.

### Data analysis

All the values generated from this study were subjected to one-way analysis of variance (ANOVA) followed by a *t* test using *R*.

## Supplementary information

Dataset 1

Dataset 2

Dataset 3
